# Radiolytic support for oxidative metabolism in an ancient subsurface brine system

**DOI:** 10.1093/ismeco/ycae138

**Published:** 2024-11-05

**Authors:** Devan M Nisson, Thomas L Kieft, Julio Castillo, Scott M Perl, Tullis C Onstott

**Affiliations:** Department of Geosciences, Princeton University, Princeton, NJ 08540, United States; Department of Biology, New Mexico Institute of Mining and Technology, Socorro, NM 87801, United States; Department of Microbiology and Biochemistry, University of the Free State, Bloemfontein, 9300, South Africa; Department of Earth, Planetary, and Space Sciences, University of California Los Angeles, Los Angeles, CA 90095, United States; Mineral Sciences, Los Angeles Natural History Museum, Los Angeles, CA 90007, United States; Blue Marble Space Institute of Science, Seattle, WA 98104, United States; Department of Geosciences, Princeton University, Princeton, NJ 08540, United States

**Keywords:** hypersaline brine, subsurface biosphere, single-cell amplified genomes, radiolysis, microbial diversity

## Abstract

Long-isolated subsurface brine environments (Ma-Ga residence times) may be habitable if they sustainably provide substrates, e.g. through water-rock reactions, that support microbial catabolic energy yields exceeding maintenance costs. The relative inaccessibility and low biomass of such systems has led to limited understanding of microbial taxonomic distribution, metabolism, and survival under abiotic stress exposure in these extreme environments. In this study, taxonomic and metabolic annotations of 95 single-cell amplified genomes were obtained for one low biomass (10^3^–10^4^ cells/ml), hypersaline (246 g/L), and radiolytically enriched brine obtained from 3.1 km depth in South Africa’s Moab Khotsong mine. The majority of single-cell amplified genomes belonged to three halophilic families (*Halomondaceae* (58%), *Microbacteriaceae* (24%), and *Idiomarinaceae* (8%)) and did not overlap with any family-level identifications from service water or a less saline dolomite aquifer sampled in the same mine. Functional annotation revealed complete metabolic modules for aerobic heterotrophy (organic acids and xenobiotic oxidation), fermentation, denitrification, and thiosulfate oxidation, suggesting metabolic support in a microoxic environment. Single-cell amplified genomes also contained complete modules for degradation of complex organics, amino acid and nucleotide synthesis, and motility. This work highlights a long-isolated subsurface fluid system with microbial metabolism fueled by radiolytically generated substrates, including O_2_, and suggests subsurface brines with high radionuclide concentrations as putatively habitable and redox-sustainable environments over long (ka-Ga) timescales.

## Introduction

The terrestrial subsurface biosphere harbors a large portion of prokaryotic life (estimated at 13–55% of total prokaryotic biomass on Earth and 18%–85% of global (terrestrial and marine) subsurface prokaryotic biomass, ~10^30^ cells), with cellular densities exhibiting significant decline along depth gradients [1]. This trend of decreasing microbial biomass at depth is accompanied by decreasing surficial redox and carbon input [2, 3]. Consequentially, many communities at depth are dominated by anaerobic, chemolithotrophic metabolisms that can be sustained by *in situ* water-rock generation of redox species and intracommunity carbon recycling [1, 4–11]. The decay of local radionuclides (e.g. ^238^U, ^232^Th, and ^40^K) from host rock and subsequent splitting of water into primary (OH-, H_2_O_2_, H_2_) and secondary (O_2_) products, known as water radiolysis, is one such water-rock process that has been suggested to sustain subsurface microbial life [12–18]. Previous work on radiolytically supported microbial life highlights the ability of this reaction to produce H_2_ for use as an energy source by chemolithotrophic metabolisms [12–18].

In addition to supplying electron donors like H_2_, water radiolysis can lead to the oxidation of inorganic species, generating electron acceptors (e.g. O_2_, NO_2_^−^/NO_3_^−^, Fe^3+^, SO_4_^2−^) for use in anaerobic and microaerophilic strategies like methanogenesis, denitrification, sulfate reduction, or acetogenesis [19]. Further, radiolytically produced oxidant species, such as O_2_ could be used by aerobic microorganisms in deep subsurface environments that would otherwise receive little to no oxygen input, even in the presence of high H_2_ concentrations [20]. Although O_2_ is a highly favorable metabolic electron acceptor, increased radiolytic activity may also lead to increased microbial oxidative stress response, due to the production of reactive oxygen species (OH^−^, H_2_O_2_), with some microorganisms shown to increase DNA repair activity once ionizing dosages exceed terrestrial background (3 mGy/yr) [21–23]. Thus, if radiolytic O_2_ is to benefit microbes, a low radiolytic oxidant contribution for microbial catabolism must energetically outweigh the stress of radionuclide decay. Current considerations of microbial radiolytic oxidant species utilization, however, primarily consider subsurface environments in marine or planetary settings [24, 25] with no consideration to date of microbial habitability and survival in radiogenically enriched environments of the terrestrial subsurface.

In this study, we conducted microbial taxonomic and metabolic characterization of a novel deep, hypersaline, high-temperature, organic-rich, and highly radiogenic brine. This included samples taken from 2.9 km depth and 3.1 km depth of the 95- and 101-levels, respectively, located in Moab Khotsong mine of the Witwatersrand Basin, South Africa. Previous noble gas dating for this system revealed an ancient (1.2 Ga) subsurface residence time for the Moab Khotsong brine along with the highest radiogenic excess of ^86^Kr ever reported for free fluids [26]. This work placed Moab Khotsong alongside the brine of Kidd Creek mine in the Canadian Shield as one of two subsurface fluid systems with a residence time exceeding 1.0 Ga, but with higher levels of local radiolysis (1–100 μg/g estimated ^238^U contribution for Moab Khotsong versus 2 μg/g maximum contribution at Kidd Creek [27]) contributing to the availability of redox species and potential abiotic hydrocarbon formation [28,29]. Estimates of *in situ* generated Δ^14^C for this system suggest that the mildly oxidizing conditions (Eh of 135–161 mV SHE and up to 4.69 10–2 mmol/L dissolved O_2_) of the Moab Khotsong brines did not originate from fluid mixing or air exposure [29]. Previous evidence for *in situ* generation of alternative electron acceptors (NO_2_^−^, NO_3_^−^, SO_4_^2−^) at concentrations capable of supporting chemotrophic metabolism argues for further study of radiolytically supported microbial life in the terrestrial subsurface in radionuclide enriched regions like fractured rock fluid systems of Moab Khotsong [28].

This study aimed to characterize microbial life inhabiting the unique conditions of the Moab Khotsong brines and to determine whether a low biomass microbial community is able to be sustained in a long-isolated radiogenically enriched fluid system, despite extended exposure to radiolytic stress. The objectives were to: (i) identify major taxonomic and metabolic populations of the Moab Khotsong microbial community and (ii) evaluate microbial survival under highly radiolytic conditions of Moab Khotsong. This study additionally evaluated metagenomic community data for higher biomass, less saline, and karstic dolomite fluid from the same mine, as well as mine service water, to determine if brine microbial members may be the result of fluid mixing or drilling contamination. The novel contributions of this study include identifications of fracture fluids with high levels of radiolysis as self-sustaining habitable environments on Earth, in addition to considerations of similarly radiogenic regions in the subsurface of other planetary systems as potential targets for microbial life detection.

## Materials and methods

### Sampling sites and borehole features

Samples were collected from Moab Khotsong gold and uranium mine, located in the Klerksdorp mining district of South Africa’s Witwatersrand Basin. Hypersaline brines were sampled from 2.9 km (95-level) and 3.1 km (101-level) depth. Both brine sampling sites lie within the West Rand Group of the Witwatersrand Supergroup stratum, which is primarily composed of 2.90 Ga quartzite [30]. A less saline, paleo-meteoric fluid was sampled from a karstic aquifer in the same mine at 1.2 km depth (1200-level) within the Transvaal Supergroup that is dominated by 2.20–2.50 Ga dolomites [31]. Additionally, mine service water, which is derived from the dolomite aquifer and circulated throughout the mine, was collected from a tap on the 95-level for contamination comparison, as this fluid is used for drilling and cleaning purposes throughout the mine. Both the 1200-level dolomitic fluid and 101-level brine were sampled using stainless steel multi-port manifolds, and brine was sampled from the 95-level with a downhole U-tube and packer device [32] per the methods of Nisson *et al.* [28]. Physicochemical parameters, major inorganic and organic aqueous species compositions, are included in supplementary material Table S1.

### Cell counts

Samples designated for microbial cell counts consisted of unfiltered fluid collected into sterile 50-ml Falcon tubes and preserved with 0.2 μm-filtered formaldehyde (Sigma Aldrich, St. Louis, MO, USA) to a final concentration of 3.7%. Formaldehyde-fixed samples were stored at 4°C immediately after sampling, transferred to Princeton University in an ice-containing cooler, and subsequently stored at 4°C until use. Additional samples used for optical-based cell counts consisted of unfiltered water collected into 1-L amber bottles that had been previously combusted at 450°C overnight. These unfixed samples were immediately frozen on surface, transported in a liquid N_2_-charged dry shipper to Princeton University, and subsequently stored at −20°C. Cells were concentrated and stained with SYTO**-**9 green fluorescent dye (Thermo Fisher Scientific, Waltham, MA, USA). Cells from the service water, 101 and 1200-levels were enumerated (*n* = 3) taking the average of 30 fields of view using epifluorescence microscopy (Olympus BX60; Olympus America Inc., Melville, NY, USA) at 1000× magnification (100× UPlanApo objective lens; Olympus, Japan). Cell concentrations in the 95-level brine sample were too low to be consistently observed by epiflourescence microscopy and instead were measured by optical microscopy (viewing window of 60 μm × 120 μm with 50× objective). All organisms exhibiting non-Brownian motion within a 5-μl aliquot of each sample (*n* = 5 for 95-level) were counted as microbial cells. These represent repeat counts on separate aliquots of fluid sample from those originally presented for the same sites by Nisson *et al.* [28].

### Metagenome amplified genome (MAG) DNA extraction and sequencing

For metagenomic analyses, fluid was passed overnight (~12 h) through a 0.2-μm pore size pleated polycarbonate GE Memtrex NY filter cartridge (1200-level sample). Considering the average of the starting and ending measured flow rates at the site (56 ml/min), ~40 L of fluid was filtered. For the service water sample, fluid was filtered through a 0.1-μm pleated polycarbonate Whatman Polycap filter (VWR International, Radnor, PA, USA) with a flow rate of ~100 ml/min for 4 h, resulting in ~42 L of fluid filtered. Upon collection, excess fluid was drained from the filters and replaced by RNAlater solution (Thermo Fisher Scientific, Waltham, MA, USA). Filters were kept in an ice-filled cooler during each underground sampling session, immediately frozen on surface, and transported in a liquid N_2_-charged dry shipper to Princeton for storage at −80°C until use. DNA was extracted from processed frozen filter samples using a custom protocol, with filter processing and extraction details included in supplementary material.

DNA was quantified for each extract using a Qubit 3.0 fluorometer with the dsDNA HS assay kit (Invitrogen, Carlsbad, CA, USA) and checked for quality of read size distribution using an Agilent 2100 Bioanalyzer (Agilent, Santa Clara, CA, USA). Libraries were prepped for extracts using the Nextera DNA Flex Library Prep kit (Illumina, San Diego, CA, USA). Constructed libraries were sequenced on an Illumina NovaSeq 6000 via a NovaSeq SP Flow Cell (2 × 150 bp mode) at Princeton University’s Genomics Core Facility. Sequencing resulted in ~300 million and ~71 million paired end reads for 1200-level and service water samples, respectively. Quality control (QC) of demultiplexed raw FASTA files was completed with fastp v.0.12.6 (Phred score > 30, >50 bp length, ≤10% bases unqualified) [33]. QC reads were assembled into contigs using the “metaspades” and “only assembler” modes in Spades v.3.11.0 [34]. QC reads were mapped back to contigs using bowtie2 v.2.3.2 (entire read alignment, very sensitive) to determine coverage [35]. Contigs were then binned using Metabat v.2.11.2 [36]. Bin quality was determined with CheckM v.1.0.7 [37]. Only high (>90% completion, <5% contamination) and medium (≥50% completion, <10% contamination) quality bins were retained [38]. A parallel extraction for contamination control was performed on RNAlater-preserved Whatman Polycap and GE Memtrex NY filters that had MQ H_2_O passed through in lieu of sample. The extraction yield did not exceed the detection limit on the Qubit fluorometer (<1 ng/μl) for the blank samples, and they were not included in sequencing analysis. While both brine samples from the 95- and 101-levels did yield extraction quantities above this Qubit detection limit, total sample quantities of 4 and 40 ng DNA, respectively, did not pass quality specifications of $\ge$100 ng DNA required for MAG sequencing at the genomics core facility. While the 95-level did not yield high enough cell counts for fluorescent cell sorting and SAG analysis ($\ge$10^4^ cells/ml), the 101-level sample was able to be processed for SAG generation.

### Single-cell amplified genome DNA extraction and sequencing

For single cell samples of the 101-level brine, 1 ml aliquots of unfiltered fluid were distributed into 2-ml polypropylene cryogenic vials with 5% glycerol and 1× TE buffer (final concentrations) at pH 8.0 at the time of sampling and then stored at −80°C until use. Single-cell amplified genome (SAG) preparation and sequencing were performed in the Single Cell Genomics Laboratory at the Bigelow Laboratory for Ocean Sciences. Cells were initially sorted via fluorescence-activated cell sorting (FACS) using an in Flux Mariner (BD) into individual wells of a 384-well plate. This technique allows separation of individual cells by selecting particles with <40 μm diameter and by detecting fluorescence corresponding to SYTO**-**9 membrane-permeating DNA stain [39]. Within each well, cells were lysed and DNA was denatured using two freeze–thaw cycles and treatment with KOH following the protocol of Stepanauskas *et al.* [39]. DNA was then amplified using the center’s own WGA-X multiple displacement amplification technique [39]. Amplifiable SAGs were sequenced using an Illumina NextSeq 2000 (2 × 100 bp mode), resulting in ~400 million paired end reads. For the 101-level brine sample, 121 wells contained individual cells capable of undergoing DNA amplification and sequencing. Resulting sequences were quality-controlled by trimming with Trimmomatic v0.32 (Phred score > 33, ≥35 bp length, remove trailing low-quality bases <5, 4-bp sliding window with quality cuts when <15) [40]. Reads with a ≥95% identity to human DNA were removed. QC reads were normalized with kmernorm v.1.05 (21 kmer size, 30 coverage, 3 cutoff; http://sourceforge.net/projects/kmernorm) and assembled with Spades v.3.11.0 [34] (careful, single cell, phred offset 33). Assembled contigs underwent further QC by trimming 100 bp from ends and retaining contigs >2000 bp in length [40]. Assembled SAG quality metrics were determined with Anvi’o v.7.1 [41] and CheckM v.1.0.7 (completeness and contamination) [37]. All SAGs with <1% completeness and/or >10% contamination were not considered further. DNA extraction and genomic sequencing were attempted on the 95-level brine sample, but neither the minimum DNA concentration for MAG generation (100 ng) nor the minimum cell density required for FACS detection and sorting into the SAG amplification workflow (~10^4^ cells/ml) were achieved. As a result, sequence-based identification for members of this community was not possible.

### Taxonomic identification and metabolic annotation

Open reading frames were identified for all MAGs and SAGs using Prodigal [42]. Taxonomic identifications were completed with GTDB-Tk release 207_v.2 [43], which employs HMMER [44] to determine bacterial and archaeal marker genes and multiple sequence alignment using pplacer [45] to determine taxonomic placement. SAGs that were not given a GTDB-Tk taxonomy assignment but showed a ≥95% average nucleotide identity (using fastANI whole genome nucleotide similarity tool [46]) to pre-annotated SAGs were assigned to the same genera. Metabolic annotations were completed with METABOLIC-C v.4.0 [47]. This program identifies metabolism-associated genes using KOFAM (KEGG database), Pfam, MEROPs, TIGRfam, and dbCan2 (CAZy) databases [48–53] and generates total community carbon, nitrogen, and sulfur cycling diagrams. Additional metabolic annotation from KEGG and CAZy databases, not captured in nutrient cycling diagrams, was determined and visualized using Anvio v.7.1 [41]. Affinities of electron transport chain (ETC) complexes, including cytochrome types, identified through KEGG annotation, were displayed using DRAM v.1.4.3 [54] and included in Fig. S3. Metabolic annotations for the 101-level brine were examined at the genus level to capture the greatest variation in metabolic contribution.

### Evaluating microbial survival under extended radionuclide decay

Microbial survival during long periods of elevated radionuclide decay was considered for model bacterial species *Escherichia coli*, *Bacillus subtilis*, *and Deinococcus radiodurans* under the site-specific dosage conditions of Moab Khotsong*.* These illustrative examples represent a range of bacteria from radio-sensitive to highly radio-resistant (*E. coli < B. subtilis < D. radiodurans*). The accumulated dosage in grays (Gy) at which a 10^−6^ population fraction remains has been determined in previous culture dosage experiments for these species. These values were extrapolated from published values to 8400 Gy for *B. subtilis* [55], as well as 1000 and 15 000 Gy for *E. coli* and *D. radiodurans* [56], respectively. The times at which these accumulated dosage values were achieved under various dosage/particle scenarios for Moab Khotsong-specific conditions were used to calculate the time of inactivity for each single-species bacterial population (i.e. time at which survival of the original population was terminated). This is similar to considerations of bacterial radiolytic survival employed in studies for Martian subsurface conditions [57,58]. A more detailed description of methods is presented in supplementary material.

**Figure 1:**
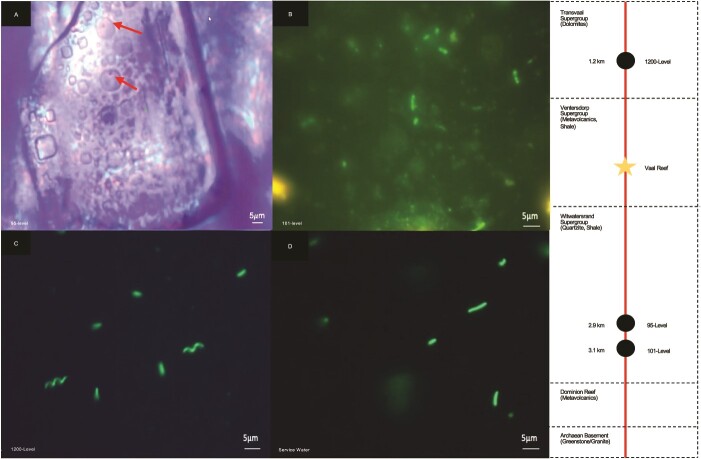
Images of cells via optical microscopy for 95-level (A), and via fluorescence microscopy combined with SYTO-9 staining for the (B) 101-level, (C) 1200-level, and (D) service water. Scale bar on the lower right of each image represents 5 μm. Magnification was 50x for (A) and 1000x for (B), (C), and (D). Cells in (A) are circular features entrapped in halite fluid inclusions and identified by arrows. Cells in (B), (C), and (D) appear as rod or spiral shaped objects. In (B), background fluorescence highlights the high level of DOC debris for this sample [28]. Cell counts for the 95-, 101-, 1200-levels and service water were 10^2^ cells/ml ± 10, 10^4^ cells/ml ± 10^2^, 10^6^ cells/ml ± 10^4.5^, 10^5^ cells/ml ± 10^3.3^, respectively (error in standard deviation). A schematic is included to the right showing the depth and major lithology of fluid sampling sites considered in this study.

## Results

### Microbial abundance

The presence of microorganisms was confirmed in all fluids sampled from Moab Khotsong mine (Fig. 1). Cells were too low in abundance to be counted in the 95-level brine via SYTO-9 staining and fluorescence visualization, but counts of 10^2^ ± 10 SD cells/ml were obtained via optical visualization of cells entrapped in halite fluid inclusions (*n* = 5) (Fig. 1A). Counts ranged up to two orders of magnitude higher for the 101-level brine (Fig. 1B, 10^4^ cells/ml ± 10^2^ SD), followed by slightly higher biomass in the service water (Fig. 1D, 10^5^ cells/ml ±10^3.3^ SD) and 1200-level fluids (Fig. 1C, 10^6^ cells/ml ± 10^4.5^ SD) (*n* = 3 for each). Counts of 10^4^ cells/ml for the 101-level brine were confirmed via FACS as part of the single-cell genomics pipeline from one submitted brine sample.

### Community taxonomic identification

**Figure 2:**
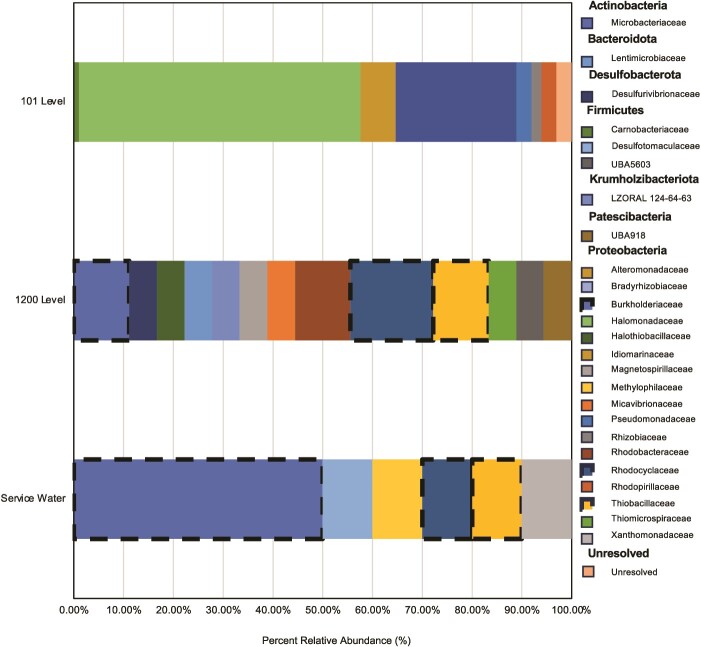
Percent relative abundance (%) of GTDB-Tk-identified families in 101-level brine SAGs, 1200-level MAGs and service water MAGs. Shared taxa between the 1200-level and service water are shown with a dashed border, which could indicate infiltration of contaminating service water microorganisms not native to the 1200-level community. Family taxonomic names are distinguished by bolded phylum-level groups in the legend.

Of the 121 single cells from the 101-level brine that underwent amplification, 95 were assembled into de novo SAGs with a CheckM estimated completion >1% and contamination <10% (Table S2). The 1200-level and service water samples yielded 18 and 10 de novo assembled MAGs (at minimum ≥50% completion and <10% contamination), respectively (Table S3). Single copy gene-based taxonomic identification by GTDB-Tk determined that all assigned taxa among the three fluid samples belonged to the Bacteria domain, with no members belonging to *Archaea* or *Eukarya*. The 101-level brine SAGs were assigned to phyla *Proteobacteria* (75%), *Actinobacteria* (24%), and *Firmicutes* (1%). For the 1200-level, the majority of MAGs were assigned to *Proteobacteria* (70%), followed by 6% of MAGs assigned to each of the phyla *Bacteroidota*, *Desulfobacterota*, *Firmicutes*, *Patescibacteria*, and *Krumholzibacteria*. Service water MAGs belonged to either *Proteobacteria* (90%) or *Firmicutes* (10%). Despite some similarities in phylum-level assignments for the 101-level brine compared to the 1200-level and service water fluids, these did not extend to the family level (Fig. 2). The 101-level brine SAGs belonged to eight different families, including: *Halomonadaceae* (58%), *Microbacteriaceae* (24%), *Idiomarinaceae* (8%), *Pseudomonadaceae* (3%), *Rhodospirillaceae* (3%), *Rhizobiaceae* (2%), *Carnobacteriaceae* (1%), and *Salinarimonadaceae* (1%). The service water displayed the lowest diversity with MAGs belonging to 6 families, but 3 of these were shared with the 1200-level, which had 13 total family-level identifications (*Burkholderiaceae* (50% service water, 11% 1200-level), *Rhodocyclaceae* (10% service water, 17% 1200-level), and *Thiobacillaceae* (10% service water, 11% 1200-level)).

### Community functional annotation: nutrient cycling and stress response

**Figure 3:**
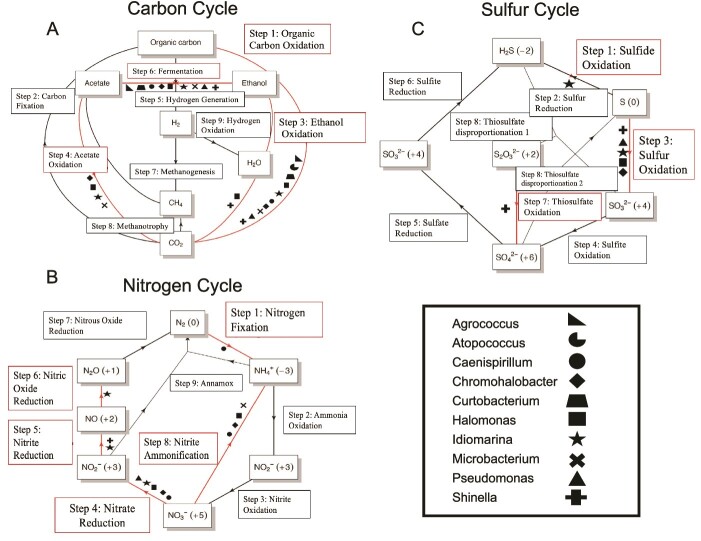
Total community nutrient cycling diagrams for 101-level brine SAGs as determined by METABOLIC-C for (A) carbon, (B) nitrogen, and (C) sulfur. Red arrows indicate the metabolic function is present in at least one SAG in the community (≥75% of gene annotations found for that function in a SAG). Symbols indicate genera associated with a particular pathway, based on individual nutrient cycling annotations for the top three most complete SAGs in each genus, including *Agrococcus* (right triangle), *Atopococcus* (three-quarter circle), *Caenispirillum* (circle), *Chromohalobacter* (diamond), *Curtobacterium* (trapezoid), *Halomonas* (square), *Idiomarina* (star), *Microbacterium* (x mark), *Pseudomonas* (equilateral triangle), and *Shinella* (plus sign).

Major metabolic pathways used by the 101-level brine community for cycling of carbon, nitrogen, and sulfur were determined with genus-level associations (Fig. 3). Heterotrophic strategies were highly favored among all SAGs (Fig. 3), including aerobic oxidation of low molecular weight organic acid species. Oxidation of acetate was specifically favored by *Chromohalobacter*, *Idiomarina*, and *Microbacterium* genera, while ethanol oxidation was favored by *Shinella*. *Halomonas* was present for all three aerobic heterotrophy pathways identified through METABOLIC-C. Additionally, all genera apart from *Atopococcus* were also capable of anaerobic fermentation, producing acetate and ethanol, suggesting the use of multiple carbon utilization pathways as a successful strategy within this community. No one SAG displayed a complete set of annotations for pathways of carbon fixation, methanogenesis, or hydrogen oxidation.

**Figure 4:**
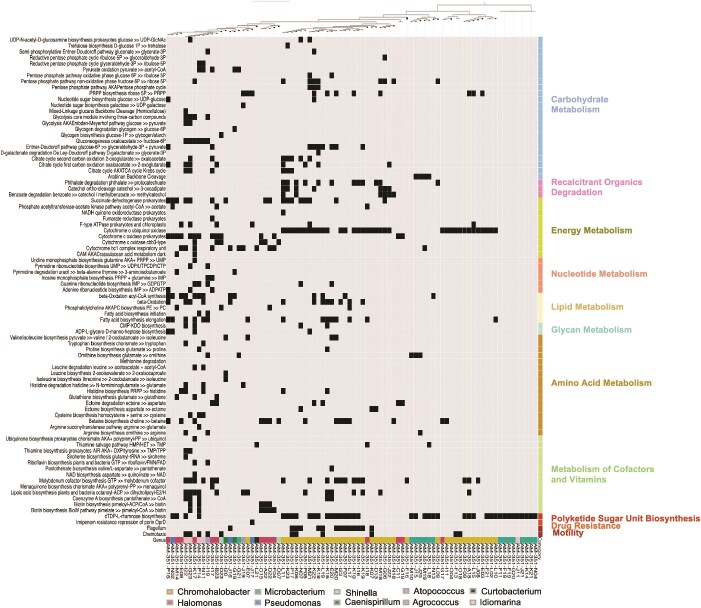
Presence/absence of KEGG metabolic modules for 101-level SAGs. Black squares indicate a module is present for a particular SAG (>70% of KOFAM annotations are present for the identified KEGG metabolic module). Colored categories on the right indicate the broader KEGG functional category for each module. SAGs are positioned in the tree relative to module presence or absence.

Nitrogen cycling strategies included denitrification, with *Idiomarina* as the only genus capable of denitrification resulting in nitrous oxide production (Fig. 3B). Other denitrifiers included *Shinella* (capable of nitrite reduction to nitric oxide) and *Halomonas*, *Pseudomonas*, *Chromohalobacter*, and *Caenispirillum* capable of nitrate reduction to nitrite. *Halomonas*, *Chromohalobacter*, and *Microbacterium* had complete pathways for nitrate ammonification. *Caenispirillum* displayed annotations for partial denitrification and ammonification, as well as being the only member capable of nitrogen fixation. Fewer genera were associated with complete sulfur cycling modules, but those with annotations favored oxidation of reduced sulfur species, including sulfide, thiosulfate, and elemental sulfur (Fig. 3C). In comparison, the 1200-level and service water communities displayed a greater variety of C, N, and S cycling strategies than the 101-level brine, including autotrophy, methanotrophy, and H_2_ oxidation associated with nitrous oxide reduction and reduction of oxidized sulfur species (S^0^, SO_4_^2−^) (Figs S1 and S2). Support for the preferred utilization of heterotrophic strategies across 101-level brine SAGs was additionally evident in the number of complete KEGG modules associated with glycolysis and oxidation steps of the citrate cycle (Fig. 4).

**Figure 5:**
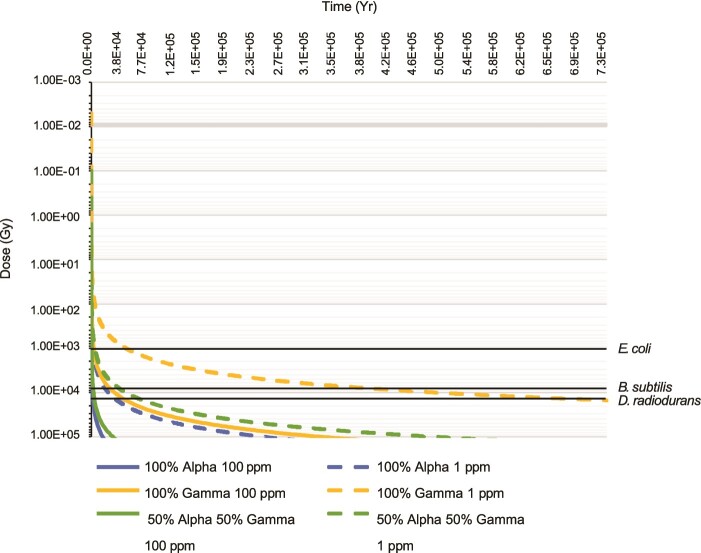
Survivability (in years) for *E. coli*, *B. subtilis*, and *D. radiodurans* under accumulated radiolytic dosage. These represent illustrative examples of how specific organisms would respond if exposed to these doses. A high radionuclide concentration scenario (100 μg/g) and a low contribution scenario (1 μg/g) were considered along with three scenarios of radionuclide type (100% alpha radiation, 100% gamma radiation, and 50% alpha with 50% gamma radiation). Black horizontal lines represent the accumulated dosage at which a 10^−6^ survival fraction is reached for *E. coli* (1000 Gy) [56], *B. subtilis* (8400 Gy) [55], and *D. radiodurans* (15 000 Gy) [56].

Complete modules for degradation of more recalcitrant substrates, including phthalates, catechol, benzoate, and arabinan, were particularly abundant within members of *Chromohalobacter* and *Halomonas*. Additionally, members of *Shinella* and *Caenispirillum* contained complete modules for glycogen synthesis and degradation, respectively, not present in other identified genera, which suggests their preference for robust carbohydrate storage and utilization. While a few members of *Idiomarina* and *Chromohalobacter* displayed hits for one portion of the reductive pentose phosphate cycle, no SAG contained a complete module encoding a full carbon fixation pathway.

In terms of energy metabolism, strong support for aerobic respiration was seen in modules in *Chromohalobacter* for lower-affinity cytochrome o ubiquinol oxidase and in cytochrome c and C/cbb3-type oxidase (*Halomonas*, *Idiomarina*, and *Pseudomonas*, *Shinella*, *Microbacterium*, and *Curobacterium*) across the vast majority of SAGs (*Atopococcus* and *Agrococcus* excluded) (Fig. 4; Fig. S3). Members of *Idiomarina* and *Halomonas* displayed several complete modules for ribonucleotide synthesis and pyrimidine degradation/biosynthesis (Fig. 4). A greater variety of SAGs included complete modules for the synthesis of amino acids alongside biosynthesis and degradation pathways of intracellular compatible solutes (osmolytes), including ectoine, trehalose, and betaine (Fig. 4). Indications of cell membrane maintenance were observed in complete fatty acid elongation and synthesis modules in most members, along with a capacity for dTP-L-rhamnose biosynthesis involved in the production of cell wall material. Motility modules associated with flagella were found among members of *Chromohalobacter*, while chemotaxis-associated motility was present in some members of *Idiomarina*, *Microbacterium*, and *Atopococccus.* In comparison to the 101-level brine SAGs, the 1200-level MAGs had a greater number of complete annotations associated with carbon fixation, but far fewer annotations associated with recalcitrant organic carbon degradation (Fig. S4). There were additionally far fewer modules associated with organic osmolyte (ectoine and betaine) biosynthesis pathways. Aerobic oxidation appeared favorable in the 1200-level MAGs as in the 101-level brine SAGs, but with a distinct preference for cytochrome c and cbb3-type complexes.

### Microbial survival under radiolytic stress

To evaluate microbial habitability under exposure to radiolysis, the survival of three single-species bacterial populations (representing a wide range of radiotolerance) was evaluated under radiolytic decay conditions of Moab Khotsong. These serve as illustrative examples of how specific organisms would respond to *in situ* dosages. Radiolytic exposure (in Gy) was calculated as an accumulated dose over time, based on either a low annual dosage scenario (0.02 Gy/yr from uranium concentrations of the West Rand (1 μg/g U)) or a high annual dosage scenario (0.3 Gy/yr with input from the uranium-enriched Vaal Reef (modeled here as 100 μg/g U) at Moab Khotsong [28]) (Fig. 5). Each bacterial population was considered inactive, or unable to survive, at the accumulated dosage resulting in a 10^6^-fold population reduction (previously determined in accumulated dosage experiments for *E. coli* (1000 Gy) [56], *B. subtilis* (8400 Gy) [55], and *D. radiodurans* (15 000 Gy) [56] cultures.

Potential microbial survival calculated based on all dosage and particle type scenarios was greatest for the radioresistant species *D. radiodurans*, followed by radiotolerant *B. subtilis*, and radio-intolerant *E. coli* (Fig. 5). The longest survival times ($\le$50 000, $\le$420 000, and $\le$750 000 years) occurred for the lower dosage (1 μg/g) scenario and gamma-type irradiation for *E. coli*, *B. subtilis*, and *D. radiodurans*, respectively. For each particle type, survival times decreased by approximately one order of magnitude from low (1 μg/g) to high (100 μg/g) dosage scenarios for each organism. For both low and high dosage scenarios, survival was shortest for more biologically damaging alpha particle irradiation (all survival $\le$5200 years for 100 μg/g, $\le$40 000 years for 1 μg/g). Consideration of equal gamma and alpha contribution still resulted in short survival times relative to 100% gamma, with all species surviving $\le$5000 years (100 μg/g) and $\le$75 000 years (1 μg/g).

## Discussion

### Cell densities and taxonomic characterization

Cell counts identified through optical and fluorescent microscopy (Fig. 1) for the different fluids followed a trend consistent with higher biomass under lower physical/chemical constraints. The lowest counts were present in the 95-level brine (10^2^ cells/ml), which has a higher temperature (55°C) but similar salinity (215 g/L) and radiolytic exposure to the 101-level brine [28], followed by higher biomass in the hypersaline 101-level brine (45°C; 10^4^ cells/ml), service water (22°C; 10^5^ cells/ml), and 1200-level dolomite fluid (26°C; 10^6^ cells/ml). In addition to having lower cell densities than the 1200-level dolomite fluid, the 101-level brine also displayed lower taxonomic diversity based on family-level identifications (Fig. 2). No taxa were shared between the two fluids, suggesting that the 101-level community may have been colonized entirely separately or has not recently received members through mixing with the dolomite fluid or meteoric waters. Additionally, even if some members of the dolomite and/or service water community were present in the brine due to current mixing, but were missed in the taxonomic annotations (e.g. as an artifact of SAG vs. MAG generation, including single cell sorting selection, low coverage sequencing, and WGA-X-based amplification used in single cell processing here [39]), then any such “contaminant” members are only a very minor portion of the current brine community. This is further supported by cellular morphology (Fig. 1) detected in fluorescent microscopy analyses of the different fluid systems, with obvious spiral shaped members of *Rhodospirillales* absent in either of the bacilli- (101-level) or cocci- (95-level) dominated brines.

Looking closer at 101-level SAG taxonomies, many of the identified genera (e.g. *Chromohalobacter*, *Halomonas*, *Microbacterium*, and *Idiomarina*) are not only found in saline and hypersaline environments [59–64], but are also capable of growth across a wide range of salinities [60,65]. Both a marine origin and salinity tolerance for many of the 101 brine SAGs support the possibility of an original community colonizing the system up to 90 Ma, when the basin cooled to biologically habitable temperatures of <150°C [66]. If the original fluid was of a more fluvial or hydrothermal origin, then it could also be that the more halophilic members were selected over time from a more diverse original community concomitant with gradual, radiolytically-driven increase in fluid salinity over that same time interval [28]. The 101-level brine did not share any taxa at the family-level or below with the service water, providing additional evidence that service water contamination is not a significant concern. The service water did share three families (*Burkholderiaceae*, *Rhodocyclaceae*, and *Thiobacillaceae*) with the 1200-level dolomite fluid from which it is sourced [67], but the low taxonomic diversity in this sample relative to both the 101 and 1200-levels is a likely artifact of the service water pipeline. The service water recirculation system includes large refrigeration- and heating-induced temperature fluctuations, in addition to enhanced radiolytic exposure from mine tailings input [67]. Additionally, standard mine wastewater treatment practices in South Africa require the application of chlorine- and bromine-based disinfectants [68], all factors that can be expected to restrict microbial habitability in this sample.

### Biogeochemical cycling strategies and support for oxidative metabolism

Functional annotation from METABOLIC-C suggests that microbial carbon cycling in the 101-level brine system is driven by heterotrophy (Fig. 3A). This includes fermentation strategies (acetate- and ethanol-producing fermentation) in addition to oxidation of acetate, ethanol, and other fermentation end products and components of the dissolved organic carbon (DOC) pool. Previous characterization of the 101-level brine highlighted that much of the organic matter for this system is part of a shale- or reef-derived kerogen pool, necessitating inhabiting microbes to access more recalcitrant substrate [29]. This capability is supported by the presence of recalcitrant organic molecule degradation gene pathways based on KEGG annotations (Fig. 4), particularly among members of *Chromohalobacter* and *Halomonas*. It is also possible that other genera lacking these recalcitrant organics degradation pathways are capable of organic matter oxidation, but prefer smaller organic molecules, including short-chain alkanes from potential abiotic production pathways, or the products of mixed acid fermentation. Several of the 101-level SAG genera are known to metabolize diverse carbon substrates [69–74], explaining why a single genus may match to multiple carbon utilization pathways (Fig. 3A).

The abundance of complete modules for cytochrome o ubiquinol oxidase alongside cytochrome c and c/cbb3-type oxidases across most brine SAGs (Fig. 4) suggests aerobic respiration as an energetically favorable alternative to anaerobic fermentation. The presence of a lower affinity type-o complex in the most abundant *Chromohalobacter* SAGs vs. preference for cytochrome c and higher affinity v/cbb3-type complexes in *Halomonas* and *Idiomarina* may indicate a tradeoff between affinity and increased flagellar motility under stressful conditions (Fig. 4; Fig. S3). This may additionally suggest a heterogenous distribution of O_2_ within the brine environment, more likely to occur if O_2_ is produced in regions localized to radionuclide-enriched sections of host rock as opposed to air exposure or mixing with toxic fluids, both scenarios that are not supported by Δ^14^C of DIC or δ^18^O for this system [29] but cannot be confirmed without δ^18^O of O_2_ [75]. Alternative terminal electron-accepting processes include the reduction of nitrate via denitrification or ammonification pathways (Fig. 3B). The availability of nitrate can be explained by the annual production of radiolytic oxidants interacting with ammonia [20,28]. There have been limited suggested pathways constraining abiotic ammonia availability in this system, with the most widely accepted being the release of NH_3_/NH_4_^+^ from surrounding phyllosilicates [19]. The microbially contributed nitrogen cycling pathways in Fig. 3B suggest, however, that ammonia could likely be recycled from radiolytically produced nitrate or produced via N_2_ fixation by *Caenispirillum*. In Fig. 3C, microbially-mediated sulfur cycling pathways are constrained to oxidation of reduced sulfur species, including thiosulfate and elemental sulfur, with members of *Idiomarina* being capable of oxidation from hydrogen sulfide. The lack of sulfate reduction capacity is consistent with the sulfide concentration being low to undetectable in the brine [28,29]. Resolving abiotic vs. biotic contribution in N and S cycling for this system will require investigation into the isotopic fractionation for these compounds.

Notably, the microorganisms of the Moab Khotsong brine did not display complete metabolic pathways for methanogenesis or H_2_ oxidation (Figs 3 and 4). While the absence of complete H_2_ oxidation gene modules does not negate a role for this metabolism in the community, it suggests this metabolic strategy is less favorable relative to other forms of oxidative metabolism. This contrasts with other less-saline fracture systems previously characterized in the Witwatersrand Basin, where support has been found for hydrogenotrophic methanogenesis in subsurface lithoautotrophic communities [5] and for high radiolytic H_2_ production and availability [14,28]. This result is, however, in agreement with micro-aerobic heterotrophic communities apparent in other global subsurface brine systems, including the Canadian and Fennoscandian Shields, further suggesting preferred utilization of radiolytically-derived oxidants in higher energy-yielding metabolic strategies under higher salinity [8,76,77] (especially if community members are synthesizing their own organic solutes, such as members using betaine and ectoine in the 101-level (Fig. 4)) and temperature stressors. This is with the exception of H_2_-driven sulfate reduction in a portion of the Kidd Creek brine community [8,76], a system where radiolytic substrates are lower [26].

Despite the favorability of aerobic metabolism as revealed through METABOLIC-C and KEGG annotations of single cell genomics, there is still the possibility that brine microorganisms subsist anaerobically on the degradation products of recalcitrant organic matter, made particularly evident by the abundance of xenobiotics degradation annotations relative to the 1200-level (Fig. 4; Fig. S4). Given the large DOC pool present in this system [29], it is difficult to determine if a smaller portion of the DOC pool is undergoing microbial turnover, potentially through fermentation and other anaerobic heterotrophic metabolic populations, as has been posited for another deep subsurface aquifer rich in buried organic C and with facultatively anaerobic members [78]. For instance, in Fig. 3A, the majority of brine SAGs do contain pathway annotations favoring fermentation strategies and all display pathways that could indicate the use of an alternative electron acceptor (e.g. oxidized N or S species, as seen in Fig. 3B–C). Even in the case that none of the excess O_2_ is used directly by the brine microbes, it is highly likely that community members are using other available redox species altered by the high levels of *in situ* radiolytic oxidation that appear limiting relative to organic matter here (Table S1). The energetic favorability of aerobic metabolism relative to anaerobic strategies [79], the presence of cytochrome genomic annotations associated with O_2_ utilization, and a consistent source of abiotically generated O_2_ all point to a deep brine community that is sustained by radiolytically supplied oxygen, either directly or indirectly via other radiolytically supplied oxidant species.

### Microbial survival under radiolysis of Moab Khotsong

Finally, the proposed radiolytic formation of the Moab Khotsong brines, alongside evidence of significant radiolytic oxidation and ^14^C production in the organic pool [29], require consideration of radiolytically imposed stress on potential *in situ* microorganisms. The diversity in radiolytic defense strategies [80], combined with a lack of experimental data on microbial maintenance under radiolytic stress, make it difficult to estimate the approximate energetic cost of radiolytic repair. It is possible, however, to consider the survival fraction for various radio-resistant bacterial species that have been previously exposed to high accumulated radiation dosages in the lab, such as *E. coli*, *B. subtilis*, and *D. radiodurans* [55,56]. Highly radio-resistant bacteria, such as *D. radiodurans*, could potentially survive up to 750 000 years if exposed to primarily gamma emission from local radionuclide concentrations of the West Rand (Fig. 5). Given that this estimate doesn’t account for actively repairing cells, this time frame could likely be extended. Additionally, radio-resistant species, such as *B. subtilis* and *D. radiodurans*, exhibit growth across broad salinities (including brine level salinities), suggesting they may be similar microorganisms to those of the Moab Khotsong brine. If introduced to the fluid system over time, such microorganisms could potentially survive under brine salinity that evolved via extended water radiolysis [28]. These same radiolytic reactions additionally provide oxidant species to the Moab Khotsong brines and are likely responsible for the availability of low oxygen levels (4.69 × 10^−2^ mmol/L) and oxidized electron acceptors, such as NO_3_^−^, in the absence of significant air-water exchange [28] or evidence for modern recharge (as supported by investigations of possible ^14^C introduction pathways by Nisson *et al.* [29]), and strong evidence for noble gas signatures supported by uranium-fission presented by Warr *et al.* [26]. The large contributions of radiolysis to the redox environment in combination with an old, radiolytically re-worked organic carbon pool suggest the potential for the Moab Khotsong brine system to host a microbial community in which anaerobic/aerobic heterotrophs could be energetically supported. Time periods of habitability ($\le$120$^{\circ}$C) have been constrained in Drake *et al.* [81] for South Africa’s Kaapvaal Craton (~650 Ma), which is shorter than maximum estimates for regions in the Canadian and Fennoscandian Precambrian Shields at ~1 Ga. These time periods suggest isolated microbial survival in these systems up to hundreds of millions of years. While definitive statements on Moab Khotsong brine community composition and survival require support from further sequence-based annotation, no other previously characterized deep subsurface fracture system in the Canadian and Fennoscandian, or South African Precambrian Shields receives such a high radiolytic dosage [13, 14, 17, 28, 82]; thus, the Moab Khotsong brine likely hosts a distinctly radiolysis-fueled oxidant-dependent heterotrophic community that has persisted in extended isolation. It is important to note, however, that these estimated survival times consider accumulated radiogenic damage in dormant cells and do not reflect survivability in metabolically active cells performing continuous repair [83]. Additionally, these estimates only reflect survival duration for these three bacterial species in isolation and do not consider the influence of survival for mixed species communities.

### Implications for habitability in radiogenic planetary environments

The presence of a low biomass, micro-aerophilic community in this long isolated and radiogenic brine system suggests that habitability in ancient planetary brines is energetically more likely in radionuclide-rich regions. Radionuclide concentrations for the most enriched regions on Mars are estimated to be lower than local concentrations at Moab Khotsong (e.g. maximum ~0.3 μg/g U on Acidalia Planitia, Mawrth Vallis [84]). It may be worth a closer look, however, at potential O_2_ production in shallower depths that receive some additional (non-lethal) contribution from ionizing radiation (from solar or galactic cosmic rays penetrating to 4.5 m depth) [85], where available water may be harbored in the Martian cryosphere and/or by hydrated clays [86–88]. This work additionally argues for increased habitability and biomarker detection in radionuclide-rich regions on putative ocean world satellites [89, 90] where high salinity brine “seas” may experience extended water radiolysis and associated production of free excess O_2_, similar to seawater radiolysis speculated to have occurred prior to the great oxidation event on early Earth [91]. Radiolytic models to further consider O_2_ production in brine environments in the subsurface of Mars and ocean worlds, including Enceladus and Europa, may be valuable to assess their habitability potential.

## Supplementary Material

SupplementaryMaterial_Final_Nisson_ycae138

Table_S_1_ycae138

Table_S_2_ycae138

Table_S_3_ycae138

## Data Availability

Raw single cell and metagenomic read data is available in NCBI’s Sequence Read Archive under BioProject numbers PRJNA946707 (Accession Numbers SRX19727896–SRX19799602) and PRJNA946159 (Accession Numbers SRX19717538–SRX19717539), respectively.
