# Supplementary material for: Radiolytic support for oxidative metabolism in an ancient subsurface brine system
Source: ISME Commun. 2024 Nov 5;4(1):ycae138. doi: 10.1093/ismeco/ycae138 (PMC11630799; doi:10.1093/ismeco/ycae138)
Supplement: SupplementaryMaterial_Final_Nisson_ycae138 [file supplementarymaterial_final_nisson_ycae138.docx]

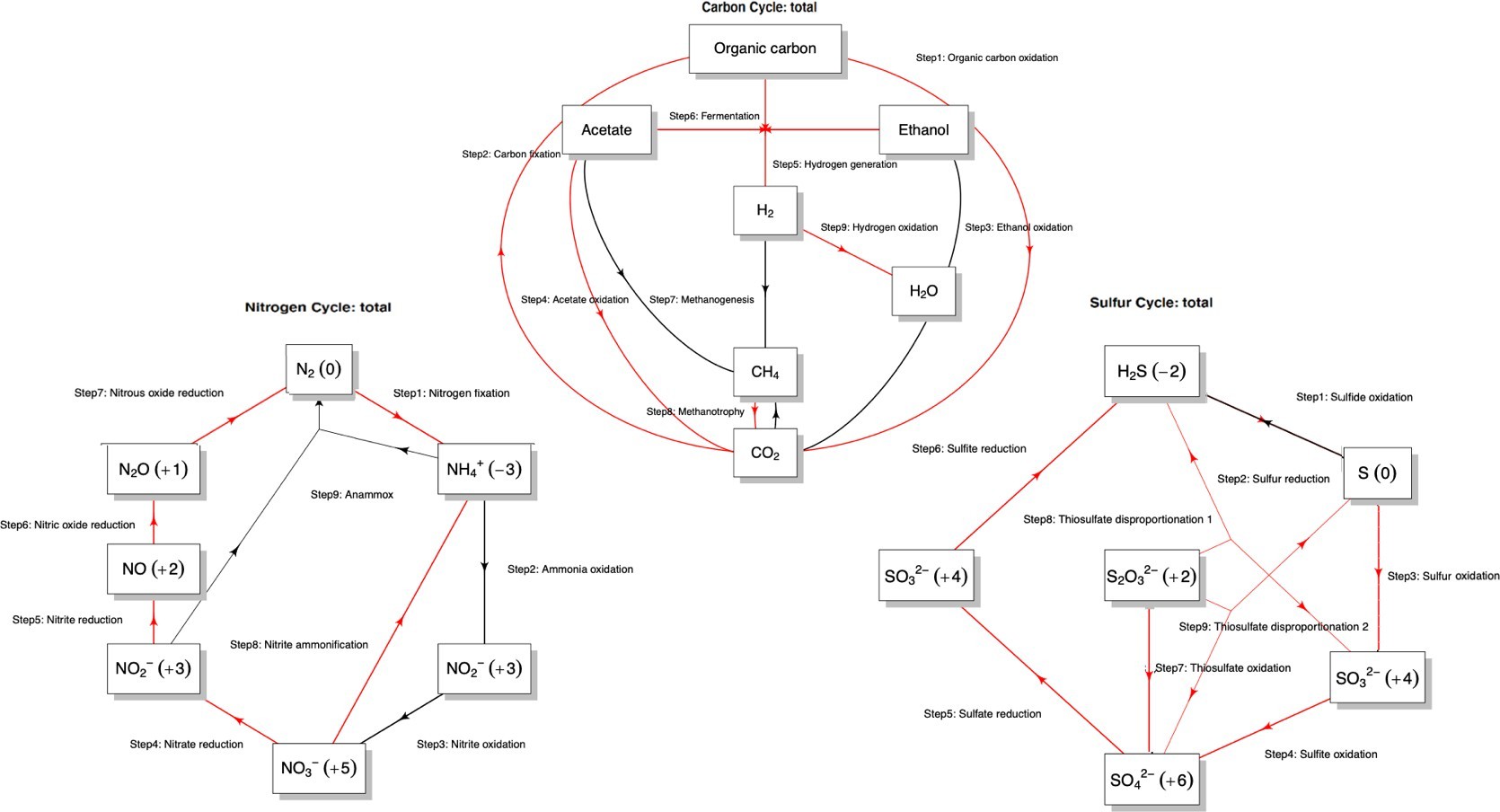
**Supplementary Material**

**Fig. S.1** Total community nutrient cycling diagrams for 1200 Level MAGs as determined by METABOLIC-C for carbon, nitrogen,

and sulfur. Red arrows indicate the metabolic function is present in at least one MAG in the community (≥75% of gene annotations found for that function in a MAG).


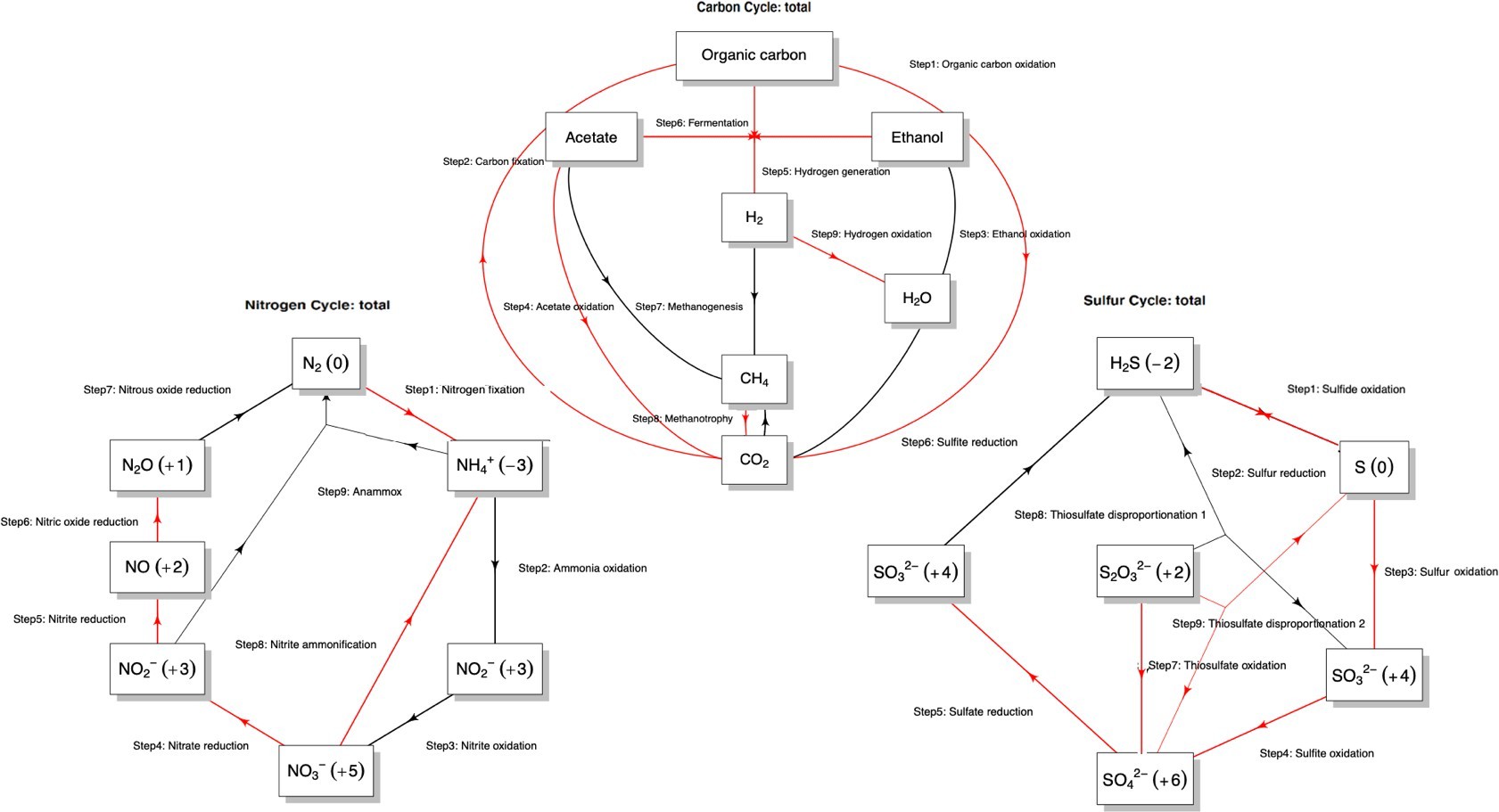


**Fig. S.2** Total community nutrient cycling diagrams for Service Water MAGs as determined by METABOLIC-C for carbon, nitrogen, and sulfur. Red arrows indicate the metabolic function is present in at least one MAG in the community (≥75% of gene annotations found for that function in a MAG).


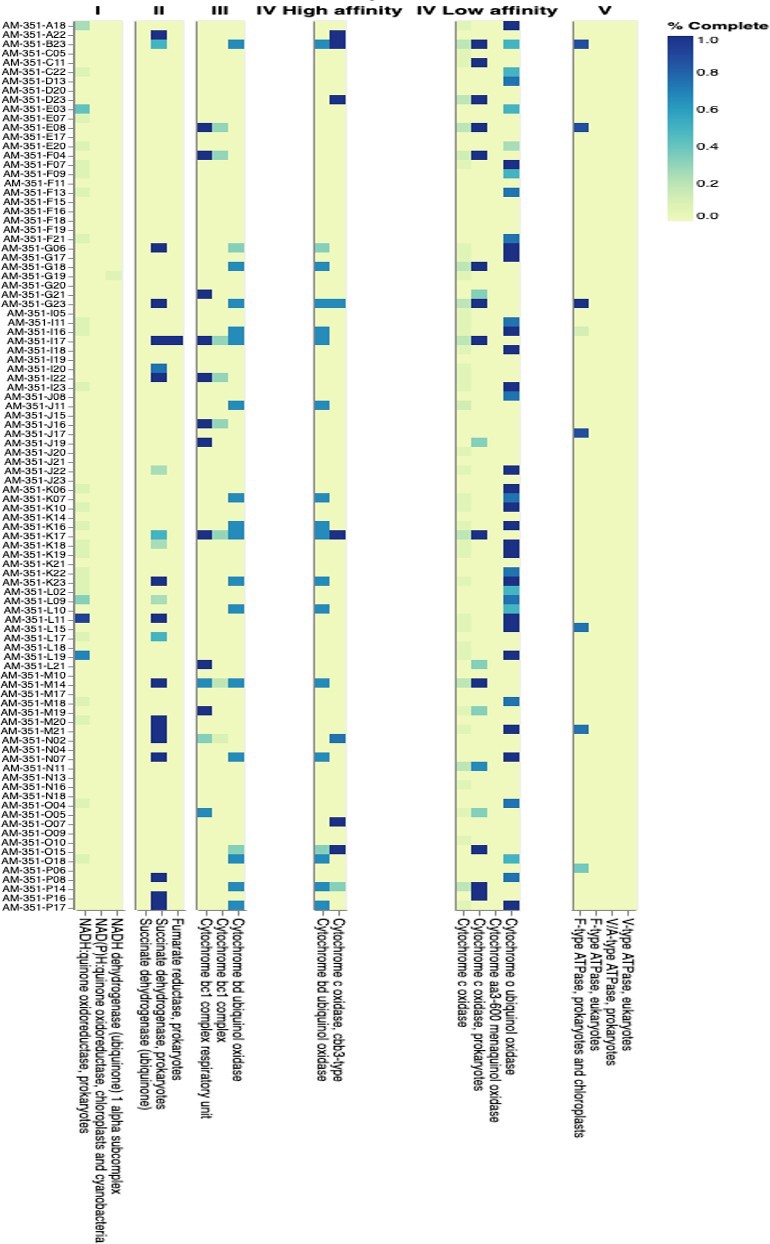


**Fig. S.3** DRAM summary metabolic module completion for 101 brine SAGs, including affinity categorization for cytochrome types (o vs. c/cbb-3 type) in electron transport chain (ETC) complexes.

**Fig. S.4** Presence/Absence of KEGG metabolic modules for 1200-level MAGs. Black squares indicate a module is present for a particular MAG (>70% of KOFAM annotations are present for the identified KEGG metabolic module). Colored categories on the right indicate the broader KEGG functional category for each module. MAGs are positioned in the tree relative to module presence or absence and are labeled with genus level identification.

**Extended Methods**

DNA Extraction Protocol

Frozen GE Memtrex NY and Polycap filters were cut into 2.5 cm sections and thawed in a horizontal clean bench (HEPA- filtered air; Labconco, Kansas City, MO, USA) [1]. The two inner sets of filter membrane were retained in 50 mL polypropylene Falcon tubes, whereas both outer and inner membranes were discarded. To each 50 mL tube, 2.5 mL of 2X CTAB lysis buffer, 250 μl of lysozyme (50 mg/ml) and 50 μl of Proteinase K (10 mg/ml) along with beating beads were added. Samples were vortexed for one minute and incubated in a water bath at 60°C for 30 minutes. The resulting liquid was transferred to a new 50 mL Falcon tube. Remaining filter pieces were rinsed with 2.5 mL of 0.2 µm-filtered MQ H_2_O and vortexed on low speed for one minute to mix. The remaining lysate was then passed through a sterile 60 mL syringe into the same 50 mL tube. To the lysate, 4 mL of phenol-chloroform- isoamyl alcohol (25:24:1) was added and samples were incubated at 60°C for one minute. Samples were transferred to ice for five minutes, and centrifuged at 17,000 x g for 10 minutes at room temperature. The remaining upper aqueous later was transferred to a 15 mL polystyrene Falcon tube, and the contents of tubes of the same sample were combined through the elution steps of a Qiagen DNeasy PowerMax Extraction Kit (Qiagen, Carlsbad, CA, USA). The resulting eluant was precipitated at -20°C overnight with two volume 100% molecular grade EtOH and 0.01 µg/µL of co-precipitant glycogen. Samples were spun at 17,000 x g and the supernatant was removed before resuspending final extracts in 200 μL 1X TE buffer.

Evaluation Microbial Survival Under Extended Radionuclide Decay

To determine if microbes could survive in the enhanced radioactivity and high radiogenic environment of Moab Khotsong (due to the high local levels of U and other elements, see below), two cases of microbial exposure to radionuclide decay were considered: (1) low 1ppm radionuclide, and a (2) high 100ppm radionuclide environment, following dosage scenarios presented in the radiolytic brine formation model of Nisson et al. [2]. These correspond to annual dosage rates of 0.02 Gy/yr and 0.3 Gy/yr, respectively. The radiolytic dosage for scenarios (1) and (2) were biologically weighted for three different cases of radionuclide composition, using corresponding particle radiation biological effectiveness [RBE] value [3]: assuming 100% alpha particle contribution, 100% gamma particle contribution, and 50% alpha with 50% gamma particle contribution. The first 100% alpha contribution case follows the assumption in Nisson et al. [2] of primarily alpha particle contribution from ^238^U; however, it is important to consider increased contribution from radionuclides other than ^238^U, as sessile microorganisms may experience radionuclide decay closer to local concentrations of the West Rand Group, in which ^232^Th (~2.5ppm), ^40^K (~0.5 %), and ^87^Rb (~14ppm) have similar or larger concentrations than ^238^U (~1ppm) [4]. Additionally, the assumption of total contribution from alpha particle radiolysis of ^238^U represents the most conservative case in brine formation [2], while in terms of

microbiology, this assumption represents the most detrimental scenario for microbial survival (RBE of 20 for 𝛼 vs. 1 for other particle types).

Microbial survival times were evaluated for three single-species bacterial populations, including *E. coli*, *B. subtilis, and D. radiodurans*, under the site-specific dosage conditions of Moab Khotsong*.* These illustrative examples represent a range of bacteria from radio-sensitive to highly radio-resistant (*E. coli < B. subtilis < D. radiodurans*). The accumulated dosage in grays (Gy) at which a 10^-6^ population fraction remains has been determined in previous culture dosage

experiments for these species. These values were extrapolated from published values to 8,400 Gy for *Bacillus subtilis* [5], as well as 1,000 Gy and 15,000 Gy for *Escherichia coli* and *Deinococcus* *radiodurans* [6], respectively. The time at which these accumulated dosage values were achieved under various dosage/particle scenarios for Moab Khotsong-specific conditions was considered the time of inactivity for each single-species bacterial population (i.e. time at which survival of the original population was terminated). This is similar to considerations of bacterial radiolytic survival employed in studies for Martian subsurface conditions [7, 8].

# **References**

1. Lau MC, Cameron C, Magnabosco C, Brown CT, Schilkey F, Grim S, et al. Phylogeny and phylogeography of functional genes shared among seven terrestrial subsurface metagenomes reveal N-cycling and microbial evolutionary relationships. *Front* *Microbiol.* 2014; 5:531.
2. Nisson DM, Kieft TL, Drake H, Warr O, Sherwood Lollar B, Ogasawara H, et al. Hydrogeochemical and isotopic signatures elucidate deep subsurface hypersaline brine formation through radiolysis driven water-rock interaction. *Geochim Cosmochim Acta*. 2023; 340:65-84.
3. Lochard J, Bogdevitch I, Gallego E, Hedemann-Jensen P, McEwan A, Nisbet A, et al. commission on radiologicalprotection. *Ann ICRP*. 2011; 120:1-125.
4. Warr O, Ballentine CJ, Onstott TC, Nisson DM, Kieft TL, Hillegonds DJ, et al. ^86^Kr excess and other noble gases identify a billion-year-old radiogenically-enriched groundwater system. *Nat. Commun*. 2022; 13:3768.
5. Baumstark-Khan C, Facius R. Life under conditions of ionizing radiation. In: Horneck G, Baumstark-Khan C (eds.) Astrobiology. Springer, 2002. pp 261-284.
6. Battista JR. Against all odds: the survival strategies of *Deinococcus radiodurans*. *Ann. Rev.Microbiol.* 1997; 51:203-224.
7. Dartnell LR, Desorgher L, Ward JM, Coates AJ. Modelling the surface and subsurface Martian radiation environment: Implications for astrobiology. *Geophys Res Lett*. 2007; 34: 10.1029/2006GL027494.
8. Teodoro L, Davila A, Elphic RC, Hamilton D, McKay C, Quinn R. Habitability and biomarker preservation in the martian near-surface radiation environment. In: Cabrol NA, Grin EA (eds.) From Habitability to Life on Mars*,* Elsevier, Pergamon, 2018. pp. 211- 231.
